# Real-world cost-effectiveness analysis of NOACs versus VKA for stroke prevention in Spain

**DOI:** 10.1371/journal.pone.0266658

**Published:** 2022-04-20

**Authors:** Carlos Escobar Cervantes, Julio Martí-Almor, Alejandro Isidoro Pérez Cabeza, Kevin Bowrin, Aleix Llorac Moix, Mar Genís Gironès, David Gasche, Aurélie Millier, Jean Tardu, Mondher Toumi, Jean-Baptiste Briere

**Affiliations:** 1 Servicio de Cardiología, Hospital La Paz, Madrid, Spain; 2 Servicio de Cardiología, Hospital del Mar, Barcelona, Spain; 3 Área del Corazón, Hospital Universitario Virgen de la Victoria, Málaga, Spain; 4 Centro de Investigación en Red de Enfermedades Cardiovasculares (CIBERCV), Madrid, Spain; 5 Bayer plc, Reading, Berkshire, United Kingdom; 6 Bayer Hispania, Barcelona, Spain; 7 Real-World Evidence Solutions, IQVIA, Madrid, Spain; 8 Creativ-Ceutical, Paris, France; 9 Aix-Marseille University, Marseille, France; 10 Bayer AG, Berlin, Germany; Karolinska Institutet, SWEDEN

## Abstract

**Aims:**

A Markov model was adapted to assess the real-world cost-effectiveness of rivaroxaban, dabigatran and apixaban. Each of these non-vitamin K antagonist oral anticoagulants was compared with vitamin K antagonist for stroke prevention in patients with non-valvular atrial fibrillation in Spain.

**Methods:**

All inputs were derived from real-world studies: baseline patient characteristics, clinical event rates, as well as persistence rates for the vitamin K antagonist treatment option. A meta-analysis of real-world studies provided treatment effect and persistence data for rivaroxaban, dabigatran and apixaban, each compared with vitamin K antagonist therapy. The model considered 3-month cycles over a lifetime horizon. The model outcomes included different costs, quality-adjusted life years and life-years gained. Sensitivity analyses were performed to test the robustness of the model.

**Results:**

When compared with vitamin K antagonist, rivaroxaban incurred incremental costs of €77 and resulted in incremental quality-adjusted life years of 0.08. The incremental cost per quality-adjusted life year was €952. For the same comparison, the incremental cost per quality-adjusted life year for dabigatran was €4,612. Finally, compared with vitamin K antagonist, the incremental cost per quality-adjusted life year for apixaban was €32,015. The sensitivity analyses confirmed the robustness of the base case results. The probabilities to be cost-effective versus vitamin K antagonist were 94%, 86% and 35%, respectively, for rivaroxaban, dabigatran and apixaban, considering a willingness-to-pay threshold of €22,000 per quality-adjusted life year gained, based on a cost-effectiveness study of the Spanish National Health System.

**Conclusion:**

These results suggest that rivaroxaban and dabigatran are cost-effective versus vitamin K antagonist for stroke prevention in non-valvular atrial fibrillation, from the Spanish National Health System perspective.

## Introduction

Atrial fibrillation (AF), the most common cardiac arrhythmia, is recognised as the primary cause of stroke, which is considered the most serious of embolic events. Patients with AF are about five times more likely to have a stroke compared with those without AF [1]. AF is the result of abnormal electrical activity disrupting the rhythm of the heart, resulting in symptoms such as chest pain, palpitations, dyspnoea, dizziness and syncope [2,3]. It is estimated to have a prevalence of 3% in the general population, which, alongside the increased risk of stroke and death associated with AF, results in significant clinical and economic burdens [4,5].

The risk of stroke and death associated with AF can be reduced with anticoagulation. Therefore, anticoagulation with a vitamin K antagonist (VKA) or non-vitamin K antagonist oral anticoagulant (NOAC) is now recognised as an important part of the treatment strategy in these patients [6–9]. There are limitations associated with VKA therapy, including variability in the effect related to clinical and/or genetic factors, drug and certain food interactions, and the need for frequent routine monitoring. Poor anticoagulation control with a VKA is associated with a higher risk of both thromboembolic and haemorrhagic complications [10]. In the last decade, NOACs have emerged as an alternative for stroke prevention in patients with non-valvular atrial fibrillation (NVAF), and clinical practice guidelines generally recommend NOACs over VKA for stroke prevention in these patients [3,11]. However, due to cost concerns, the Spanish Medicine Agency Therapeutic Positioning Report positions the NOACs as second-line therapy following VKAs, and only as a first-line therapy in certain situations [12].

Both economic and clinical evaluations are needed when Health Technology Assessment bodies make decisions regarding the reimbursement of new technologies [13–16]. Economic evaluations include cost-effectiveness models that use efficacy data to demonstrate clinical benefits, with most data originating from randomised controlled trials (RCTs) [17]. This is because RCT data are often the only data available at the time of model development, but real-world evidence (RWE) is now playing an increasingly large role in the process [14,16,18]. RWE is associated with limitations when compared with RCTs, in terms of the interpretation of results and the ability to account for potential biases, but it also offers several advantages over RCTs. For example, RWE is able to capture data on the routine care of a patient population in the real world, rather than the selected populations included in RCTs. RWE studies generally also have large sample sizes and are, therefore, able to provide different insights compared with the smaller sample sizes of RCTs. Finally, RWE can provide data on more outcomes for longer follow-up periods compared with RCTs, which usually have short- to medium-term follow-up periods and focus on a small number of outcomes [13,16]. RWE is, therefore, able to inform cost-effectiveness models on the real-world use and costs of a drug, including whether the label-recommended dose is used [14].

This paper aims to assess the cost-effectiveness of three different NOACs (rivaroxaban, dabigatran and apixaban) compared with VKA for stroke prevention in patients with NVAF in the Spanish healthcare setting, considering RWE exclusively. The results of our analysis demonstrate that, based on RWE, rivaroxaban and dabigatran are cost-effective options versus VKA for stroke prevention in NVAF from the Spanish National Health System perspective with a threshold of €22,000 per quality-adjusted life year (QALY) gained.

## Methods

### Model overview

An updated international Markov model was used to assess the cost-effectiveness of three NOACs (rivaroxaban, dabigatran and apixaban), each compared with VKA, for the first-line treatment of stroke in adult patients with NVAF and more than one risk factor for stroke [19]. The cost-effectiveness analysis was conducted from the Spanish National Health System perspective with a lifetime horizon (30 years simulated).

The latter simulates various health states based on NVAF potential complications (stable AF, acute and post-major ischaemic stroke, acute and post-minor ischaemic stroke, acute and post-myocardial infarction (MI), acute and post-intracranial haemorrhage and gastrointestinal bleeding), and the absorbing health state of death. It is assumed that during each Markov cycle of 3 months, a patient can stay within the same health state or change to a different one. Patients transition through the model, accumulating QALYs associated with each different health state, costs of pharmacological treatment, drug administration and management of clinical events. Regarding the treatment allocation, a patient can discontinue his/her initial treatment, switch from a NOAC to VKA, switch from one VKA to another VKA, or stop treatment, i.e. switch from any treatment to no treatment. The Markov model was designed to simulate long-term clinical and economic consequences up to death, or until occurrence of a subsequent event, independent of the treatment. The model outcomes included the number of different clinical events (ischaemic stroke, MI and bleeds), as well as the total QALYs, the total life-years gained (LYG), the total costs and the incremental cost per QALY or per LYG. Both health outcomes and costs were discounted at 3% per annum as recommended from the Spanish National Health System perspective [20].

### Model input parameters

Three clinical experts in stroke prevention in patients with NVAF validated the model design, the data sources (i.e. various RWE studies) and the input values used in the analysis, in a consensus meeting. All inputs are presented in Tables 1 and 2.

**Table 1:** Model inputs.

	Value	Range Used in DSA	Distribution Used in PSA	Source
3-month probabilities (VKA arm) (derived from baseline event rates per 100 patients-years)				
Minor IS	0.114%	[0.100%; 0.130%]	Beta	Weighted average of event rates identified in Giner-Soriano et al. 2017 [21] + Hylek et al. 2003 [22]
Major IS	0.163%	[0.144%; 0.187%]	Beta	
MI	0.193%	[0.181%; 0.205%]	Beta	Weighted average of event rates identified in Briere et al. 2019 [23]
GI bleeding	0.260%	[0.225%; 0.297%]	Beta	Weighted average of event rates identified in Giner-Soriano et al. 2017 [21]
ICH	0.085%	[0.072%; 0.132%]	Beta	
3-month probabilities of discontinuation				
0–3 months	15.00%	[14.19%; 15.81%]	Beta	De Andres-Nogales et al. 2015 [24]
3–6 months	10.59%	[9.89%; 11.28%]	Beta	
6–12 months	8.23%	[7.61%; 8.85%]	Beta	
12+ months	6.40%	[5.86%; 6.95%]	Beta	
**Baseline event rates per 100 patient-years (VKA arm)**				
IS	1.11	[0.98; 1.27]	-	Giner-Soriano et al. 2017 [21]
GI bleeding	1.04	[0.90; 1.19]	-	
ICH	0.34	[0.29; 0.53]	-	
MI	0.77	[0.73; 0.82]	-	Briere et al. 2019 [23]
**Proportion of switch**				
VKA	25.80%	[21.93%; 29.67%]	Beta	Johnson et al. 2016 [25]
Rivaroxaban	23.20%	[19.72%; 26.68%]	Beta	
Dabigatran	35.40%	[30.09%; 40.71%]	Beta	
Apixaban	36.70%	[31.20%; 42.21%]	Beta	
**Hazard ratio (rivaroxaban arm)**				
Minor IS	0.83	[0.75; 0.93]	Beta	Coleman et al. 2019 [26]
Major IS			Beta	
MI	0.96	[0.80; 1.14]	Beta	
GI bleeding	1.22	[1.12; 1.33]	Beta	
ICH	0.68	[0.52; 0.90]	Beta	
Discontinuation	0.62	[0.60; 0.65]	Beta	
**Hazard ratio (dabigatran arm)**				
Minor IS	0.79	[0.65; 0.97]	Beta	Coleman et al. 2019 [26]
Major IS			Beta	
MI	0.84	[0.71; 1.00]	Beta	
GI bleeding	1.12	[1.02; 1.24]	Beta	
ICH	0.45	[0.36; 0.58]	Beta	
Discontinuation	0.91	[0.53; 1.24]	Beta	
**Hazard ratio (apixaban)**				
Minor IS	1.01	[0.87; 1.17]	Beta	Coleman et al. 2019 [26]
Major IS			Beta	
MI	1.00	N/A	-	
GI bleeding	0.52	[0.38; 0.70]	Beta	
ICH	0.41	[0.28; 0.60]	Beta	
Discontinuation	1.08	[0.81; 1.45]	Beta	
**In-hospitalisation mortality rates per clinical event in the model**				
Minor IS	2.89%	[2.57%; 3.20%]	Beta	Rubio-Terrés et al. 2016 [27]
Post-minor IS	N/A	N/A	-	
Major IS	12.60%	[9.40%; 15.70%]	Beta	
Post-major IS	2.63%	[0.91%; 13.50%]	Beta	
MI	9.69%	[7.27%; 12.11%]	Beta	
Post-MI	2.68%	[0.00%; 6.75%]	Beta	
ICH	38.85%	[29.14%; 48.56%]	Beta	
Post-ICH	2.63%	[0.91%; 13.50%]	Beta	
GI bleeding	7.33%	[6.92%; 7.74%]	Beta	Ministerio de Sanidad, 2017 [28]
**Utility values**				
Stable AF	0.73	[0.71; 0.75]	Beta	Sullivan et al. 2011 [29]
Minor IS	0.73	[0.55; 0.91]	Beta	Luengo-Fernandez et al. 2013 [30]
Major IS	0.41	[0.31; 0.51]	Beta	Luengo-Fernandez et al. 2013 [30]
Post-minor IS	0.76	[0.57; 0.95]	Beta	Luengo-Fernandez et al. 2013 [30]
Post-major IS	0.56	[0.42; 0.70]	Beta	Luengo-Fernandez et al. 2013 [30]
MI	0.66	[0.53; 0.79]	Beta	Pockett 2014 et al. [31]
Post-MI	0.73	[0.58; 0.88]	Beta	Pockett 2014 et al. [31]
ICH	0.56	[0.45; 0.67]	Beta	Luengo-Fernandez et al. 2013 [30]
Post-ICH	0.67	[0.54; 0.80]	Beta	Luengo-Fernandez et al. 2013 [30]
GI bleeding	0.70	[0.56; 0.84]	Beta	Sullivan et al. 2011 [29]
**Resource use and costs (€2018)**				
**Daily treatment cost**				
VKA	0.10	N/A	-	CGCOF, 2019 [32]
Rivaroxaban	1.79	N/A	-	
Dabigatran	1.79	N/A	-	
Apixaban	1.79	N/A	-	
**IS costs**				
Acute treatment (minor)	5,258	[3,953; 6,572]	Gamma	Rubio-Terrés et al. 2016 [27] Baron-Esquivias et al. 2015 [33]
Acute treatment (major)	7,208	[5,406; 9,010]	Gamma	
Monthly follow-up (minor)	124	[93; 156]	Gamma	
Monthly follow-up (major)	2,159	[1,619; 2,699]	Gamma	Rubio-Terrés et al. 2016 [27] Hervas-Angulo et al. 2006 [34]
Rehabilitation	3,015	[2,261; 3,768]	Gamma	Rubio-Terrés et al. 2016 [27] Restovic et al. 2012 [35]
**MI**				
Acute treatment (one event per cycle)	5,174	[3,880; 6,467]	Gamma	Rubio-Terrés et al. 2016 [27]
Monthly follow-up	516	[129; 215]	Gamma	Escolar-Albaladejo et al. 2016 [36] Mar et al. 2011 [37]
**Bleeding**				
Acute treatment GI bleeding (non-ICH)	3,579	[2,685; 4,474]	Gamma	Escolar-Albaladejo et al. 2016 [36] Monreal et al. 2009 [38]
Acute treatment—ICH	7,793	[5,845; 9,741]	Gamma	Rubio-Terrés et al. 2016 [27] Baron-Esquivias et al. 2015 [33]
Monthly follow-up	191	[143; 238]	Gamma	Alvarez-Sabin et al. 2017 [39]
Rehabilitation	2,874	[2,155; 3,592]	Gamma	Rubio-Terrés et al. 2016 [27] Restovic et al. 2012 [35]
**Resource use for rehabilitation**				
% of rehabilitation for minor IS	5.0%	[4.25%; 5.75%]	Beta	Experts committee
% of rehabilitation for major IS	37.4%	[31.79%; 43.01%]	Beta	
% of rehabilitation for GI bleeding	10.6%	[9.01%; 12.19%]	Beta	
% of rehabilitation for ICH	45.0%	[38.25%; 51.75%]	Beta	

DSA, deterministic sensitivity analysis; GI, gastrointestinal; ICH, intracranial haemorrhage; IS, ischaemic stroke; MI, myocardial infarction; PSA, probabilistic sensitivity analysis; VKA, vitamin K antagonist.

**Table 2:** Relative risk for ischaemic strokes by age group [40].

Age Group	Relative Risk
55–59	0.667
60–64	0.760
65–69	0.854
70–74	1.000 (reference)
75–79	1.146
80–84	1.281
85–89	1.480
90+	1.719

#### Patient population

The analysis considered patients with characteristics drawn from a recent Spanish RWE study in order to ensure generalisability to the NVAF population in Spain [21]. The mean age of the patient population at model entry was 73.4 years, of which 51.7% were male. Moreover, 17.8% had an intermediate CHA_2_DS_2_-VASc score (0–1) and 82.2% had a high CHA_2_DS_2_-VASc score (≥2).

#### Clinical event rates

The baseline 3-month probabilities of the VKA arm were derived from existing Spanish RWE studies, which provided event rates for ischaemic stroke, gastrointestinal bleeding and intracranial haemorrhage [21]; no Spanish input was retrieved for MI, so an existing systematic review was considered [23]. The split between minor and major ischaemic stroke was derived from another RWE study conducted in the US [22]. Moreover, the risk for minor and major ischaemic stroke was adjusted by age using results from the RWE Framingham Heart Study, in order to correctly reflect the increased stroke risk, positively related with the age of the simulated patient cohort [40]. The treatment effect for each NOAC was taken from a published meta-analysis, providing hazard ratios (HRs) for all comparators versus VKA, considering RWE in both prevalent and incident populations [26].

#### Discontinuation

As a patient can remain on initial treatment or discontinue in real life, the model was updated to account for discontinuation. The discontinuation risk is unlikely to be constant over time, so the model has been adapted to capture the evolution of the discontinuation with time [25]. Discontinuation was split into four periods in the model: from initiation to 3 months, from 3 months to 6 months, from 6 months to 1 year, and after 1 year. A Spanish study reported persistence rates for VKA for two periods [24], i.e. from 3 months to 6 months and from 6 months to 1 year. Assumptions were made to calculate the 3-month probabilities of discontinuation. The comparative treatment effect for each NOAC assessed in the model, compared with VKA, was taken from the previously mentioned meta-analysis [26].

#### Mortality

Owing to the high age of the population at model entry, a background mortality rate was applied to each health state extracted from the Spanish mortality tables [41]. In addition to the mortality of the general population, a specific mortality related to each clinical event was considered [27,28].

#### Utility

As too few Spanish utility values were available, only utility values from UK studies were considered [29–31,42]. It has to be noted that no treatment-related utility decrements were considered in the base case analysis.

#### Healthcare resource use and costs

The current analysis considered drug acquisition, administration, VKA monitoring costs and costs associated with the management of clinical events. All healthcare resource use and costs were collected from Spanish RWE studies [27,33–36,39]. All these costs were updated to 2018 values according to the Consumer Price Index [43]. Expert opinion was considered for the proportion of rehabilitation after stroke or bleeding (intracranial haemorrhage or gastrointestinal bleeding).

#### Sensitivity analyses

In order to test the impact of variations in the parameters included in the model, a deterministic sensitivity analysis was conducted. Similarly, a probabilistic sensitivity analysis was performed to evaluate the parameter uncertainty on the cost-effectiveness results. For this analysis, it was assumed that the 3-month probabilities, proportion of switch, HRs, mortality rates, utilities and resource use for rehabilitation would be adjusted to beta distributions (parameter 0 to 1), and that resource use and costs would be adjusted to gamma distributions (0 to infinity). In addition, several specific scenarios were considered. The first scenario considers another source of RWE population characteristics with older patients (77 years old) and a more severe CHA_**2**_DS_**2**_-VASc mean score [44]. The second scenario tested different time horizons (10 and 20 years). Finally, a third scenario considered treatment effect, in terms of event reduction, based on international and Spanish RWE studies (Table 1) [26,45,46].

## Results

The results for rivaroxaban, dabigatran and apixaban each compared with VKA are presented in Table 3.

**Table 3:** Model results.

Outcome	VKA	Rivaroxaban		Dabigatran		Apixaban	
	Value	Value	Incr. vs VKA	Value	Incr. vs VKA	Value	Incr. vs VKA
**Costs (€)**							
Drug acquisition costs	145	2,711	2,566	2,024	1,879	1,741	1,596
Drug administration costs	1,345	928	–417	917	–427	887	–458
Clinical event management costs	18,818	16,745	–2,073	17,667	–1,151	18,634	–184
Total costs	20,307	20,384	77	20,608	300	21,262	955
**Health benefits**							
Total QALYs	7.16	7.24	0.08	7.23	0.07	7.19	0.03
Total LY	9.96	10.06	0.09	10.04	0.08	10.00	0.04
Ischaemic strokes	0.29	0.26	–0.03	0.28	–0.02	0.29	0.00
Myocardial infarctions	0.14	0.12	–0.02	0.13	–0.01	0.14	0.00
ICH	0.02	0.02	0.00	0.01	–0.01	0.01	–0.01
GI bleeding	0.05	0.07	0.02	0.06	0.01	0.04	–0.01
**Incremental costs-effectiveness ratios (€)**							
Incremental cost/QALY			952		4,612		32,015
Incremental cost/LYG			828		3,800		24,572

Data are rounded to the nearest € for costs and to two decimal places for health benefits.

GI, gastrointestinal; ICH, intracranial haemorrhage; LY, life year; LYG, life-year gained; QALY, quality-adjusted life year; VKA, vitamin K antagonist.

Patients treated with rivaroxaban experienced incremental gains in both QALYs (0.08) and life-years (0.09) compared with VKA. Patients receiving rivaroxaban experienced fewer MIs and a lower rate of strokes and intracranial bleeds, but also experienced a higher rate of gastrointestinal bleeds. However, these incremental differences between rivaroxaban and VKA were minimal. The final benefits were translated into an incremental cost-effectiveness ratio (ICER) of €952 per QALY gained and an ICER of €828 per LYG. The ICER for dabigatran versus VKA was €4,612 per QALY gained, and the ICER for apixaban versus VKA reached €32,015 per QALY gained. For dabigatran, incremental gains in both QALYs (0.07) and life-years (0.08) compared with VKA were observed, while QALY gains of 0.03 and LYG 0.04 were observed for apixaban.

Rivaroxaban is, therefore, associated with the lowest incremental cost and the highest effectiveness in terms of LYG and QALY gained versus VKA, mainly due to a reduction in stroke rate which resulted in a lower ICER versus VKA in comparison with dabigatran and apixaban. These results suggest that rivaroxaban is the most cost-effective option versus VKA.

**Fig 1:**
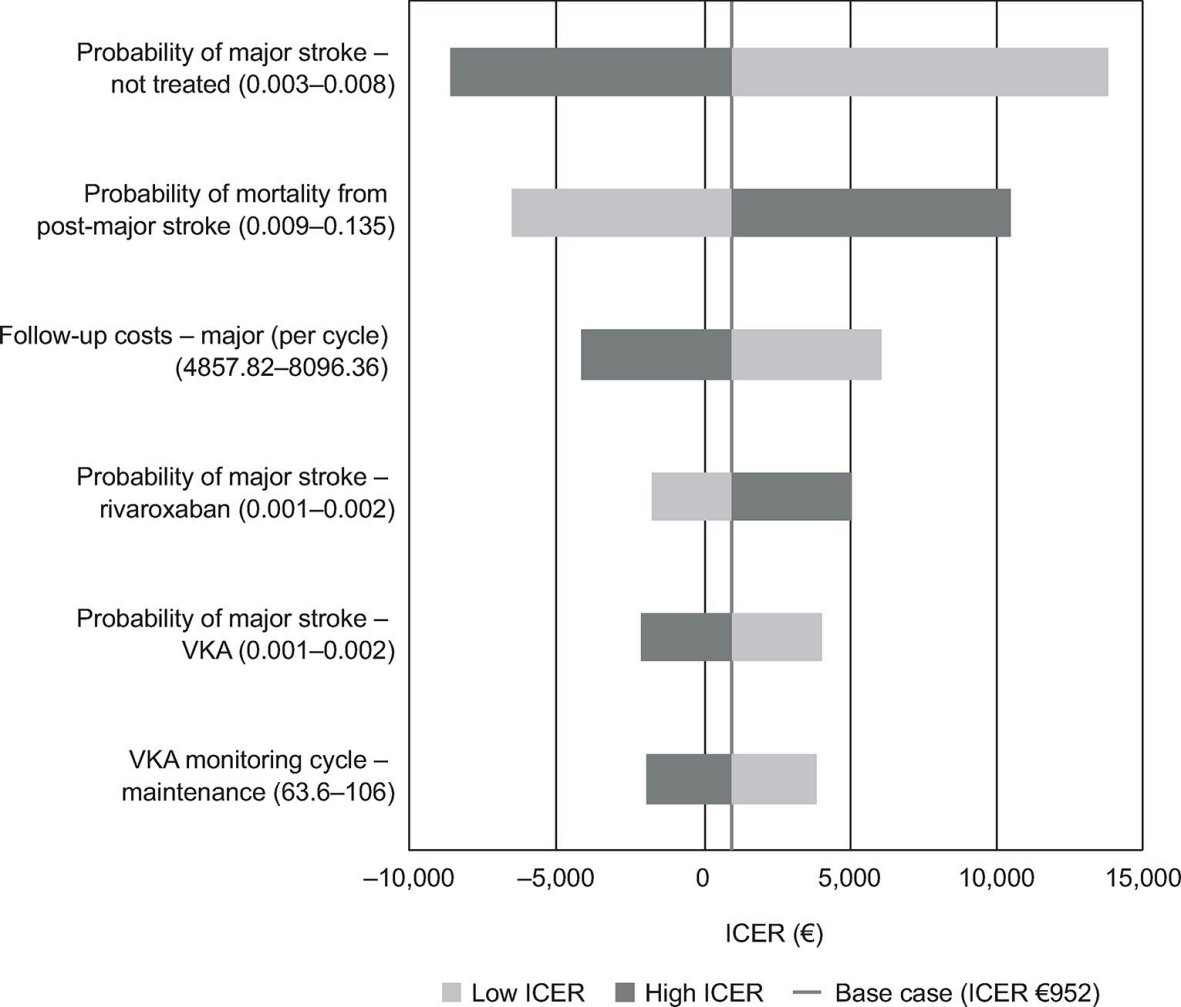
Rivaroxaban tornado diagram. ICER, incremental cost-effectiveness ratio; VKA, vitamin K antagonist.

**Fig 2:**
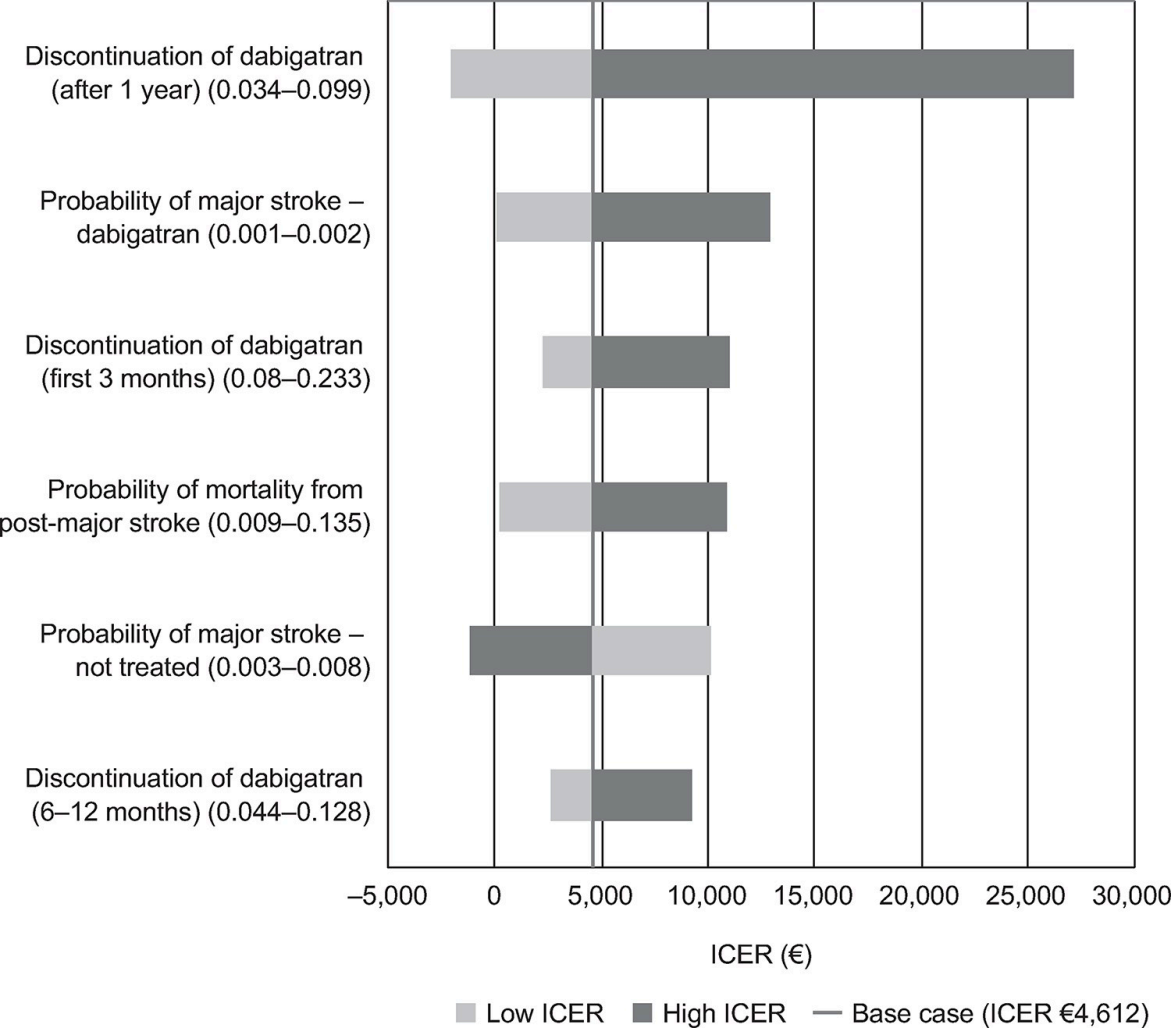
Dabigatran tornado diagram. ICER, incremental cost-effectiveness ratio; VKA, vitamin K antagonist.

**Fig 3:**
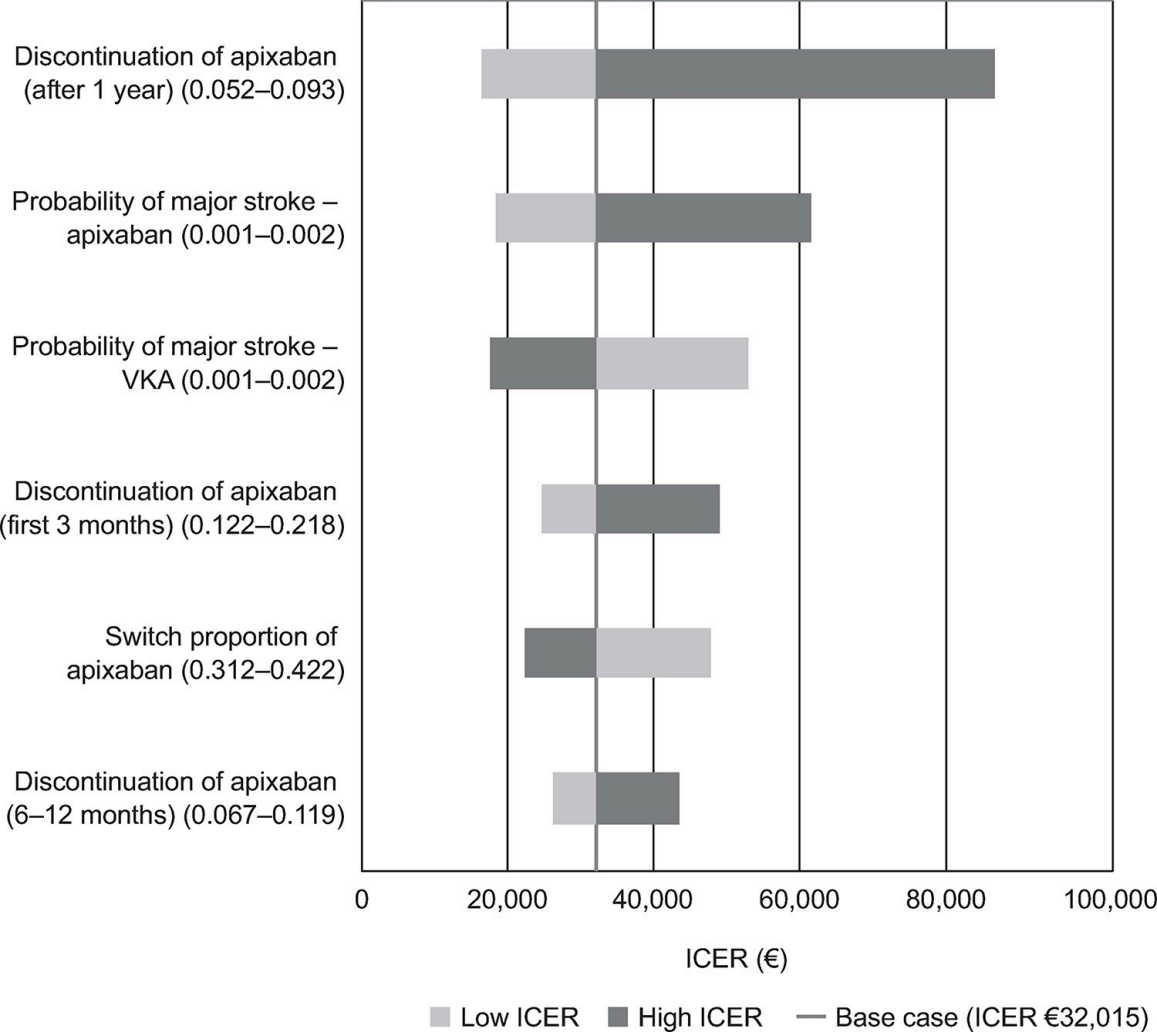
Apixaban tornado diagram. ICER, incremental cost-effectiveness ratio; VKA, vitamin K antagonist.

The main drivers of the ICERs for rivaroxaban, dabigatran and apixaban, each compared with VKA, are presented in Figs 1–3. The Tornado diagrams show that the results were robust to plausible changes in the parameter values; some parameters cross into negative ICER values, showing that the model is sensitive to the choice of parameter value. The main drivers identified were major stroke and post-major stroke mortality probabilities, as well as major stroke follow-up costs for the analysis of rivaroxaban; for dabigatran, the main drivers were dabigatran maintenance, major stroke and post-major stroke mortality probabilities; in the apixaban analysis, maintenance, major stroke probability and switch proportion were identified as the main drivers. Further studies should be performed to better pinpoint the exact value of the parameters that drive the ICERs for rivaroxaban, dabigatran and apixaban.

**Fig 4:**
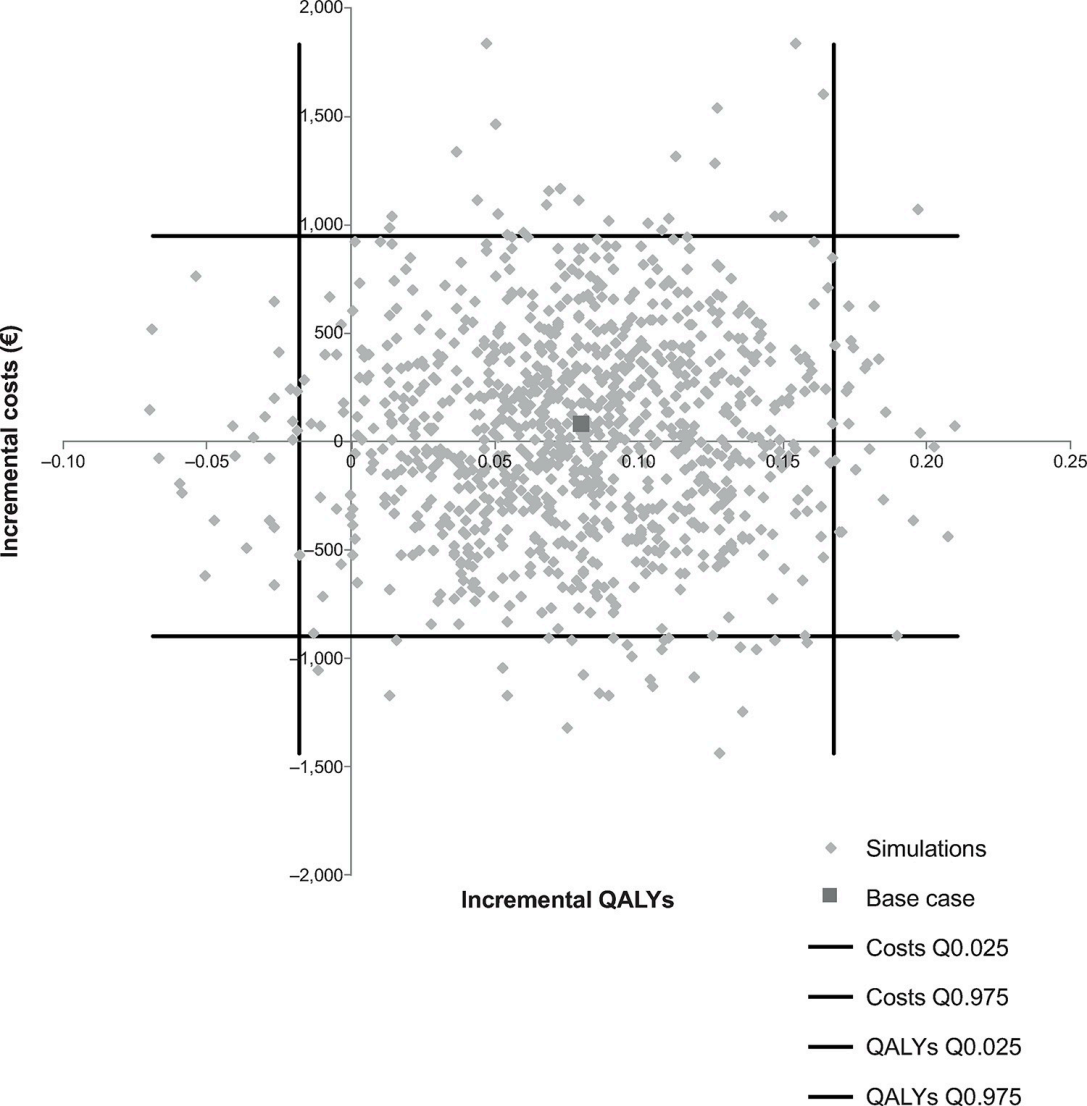
Rivaroxaban incremental cost-effectiveness plane. QALY, quality-adjusted life years.

**Fig 5:**
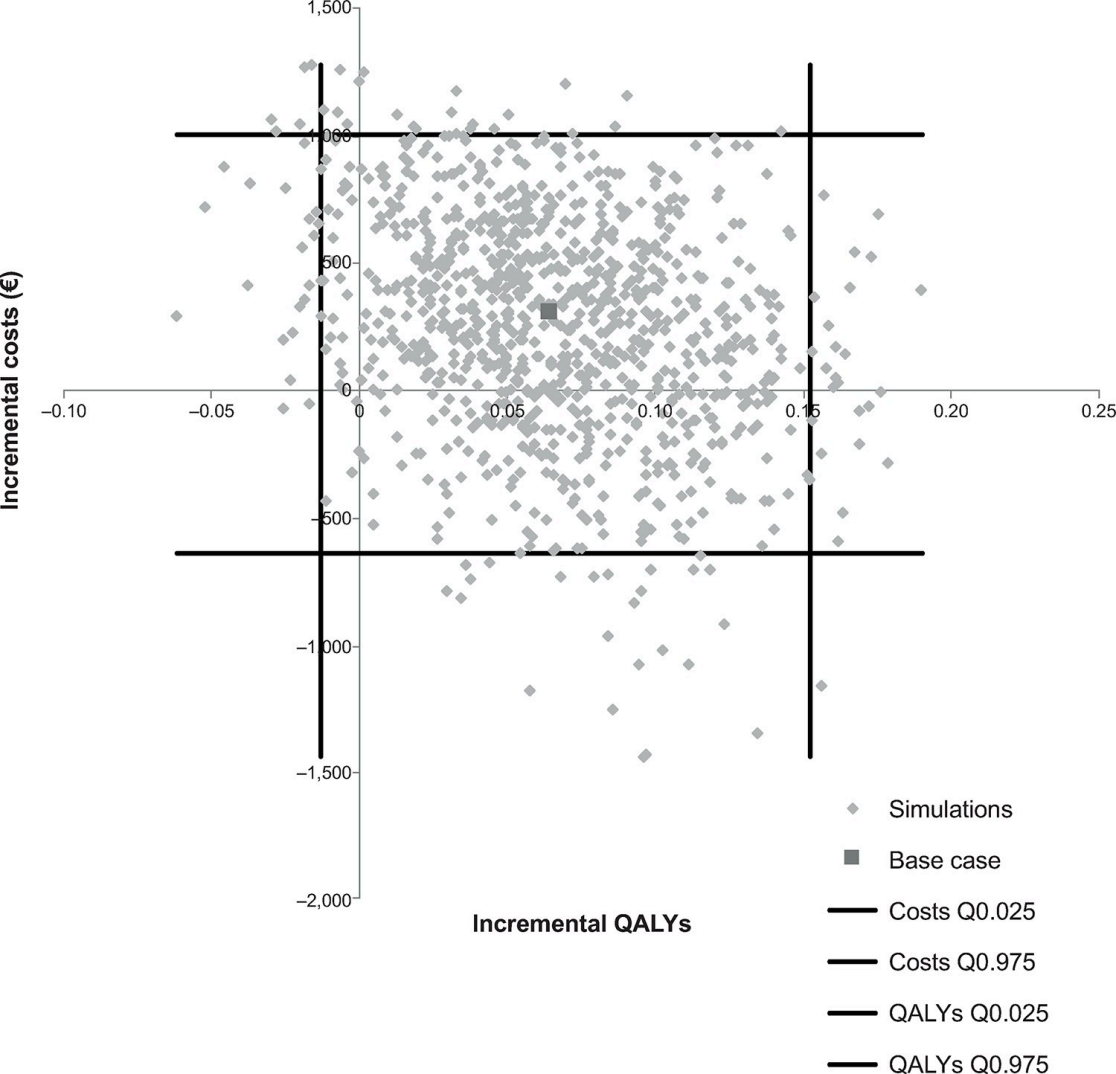
Dabigatran incremental cost-effectiveness plane. QALY, quality-adjusted life years.

**Fig 6:**
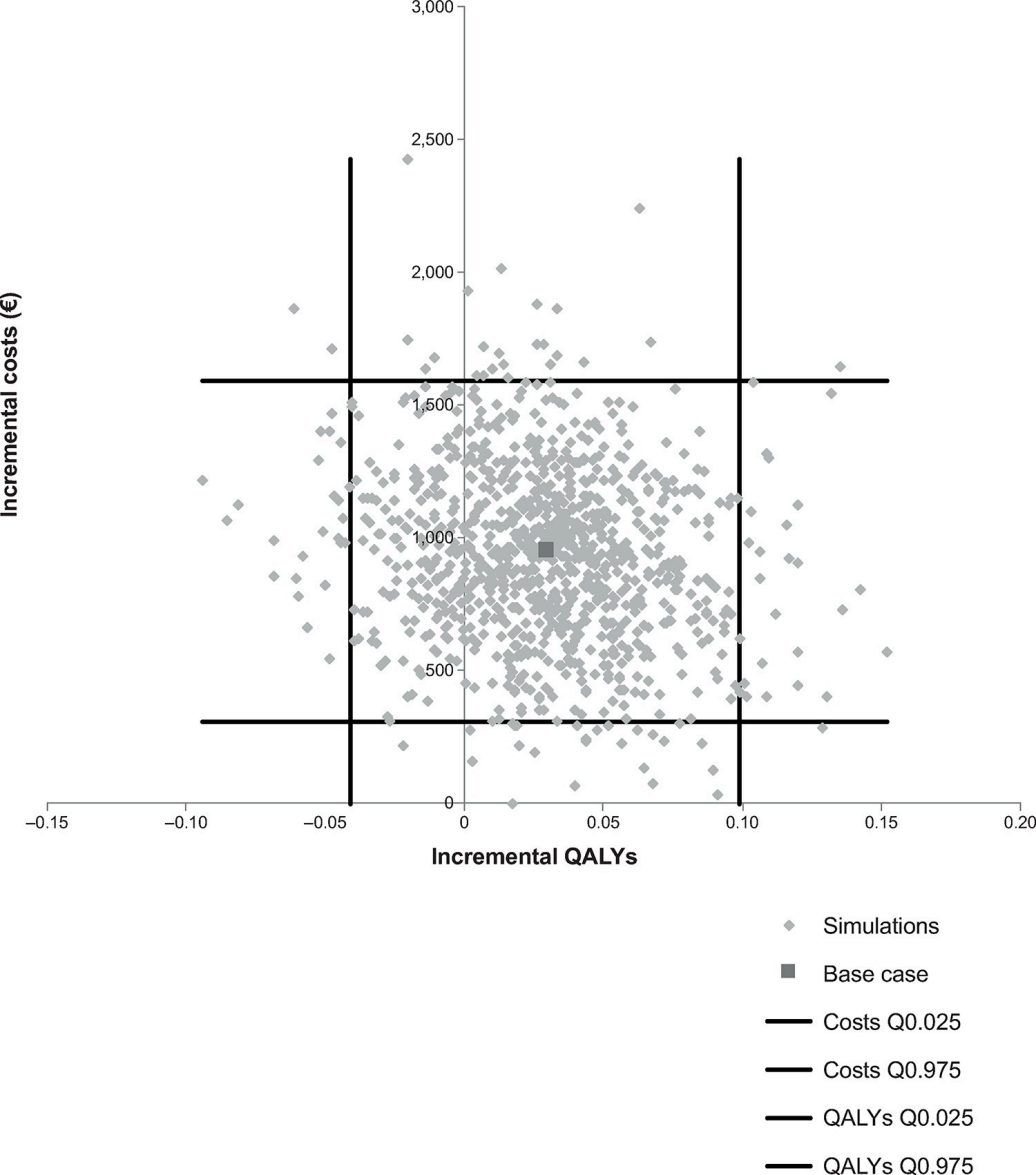
Apixaban incremental cost-effectiveness plane. QALY, quality adjusted life years.

Cost-effectiveness scatterplots for rivaroxaban, dabigatran and apixaban, each compared with VKA, are presented in Figs 4–6. There was no evidence of a correlation between incremental costs and incremental effects

No official threshold for cost-effectiveness is available in Spain, but a recent reference suggests considering a range between €22,000 and €25,000 per QALY [47]. The probabilities for each assessed NOAC to be cost-effective compared with VKA, considering a cost-effectiveness threshold of €22,000 per QALY, were 94%, 86% and 35% for rivaroxaban, dabigatran and apixaban, respectively.

The results of the scenarios are presented in Table 4. The first scenario considering another source of RWE population characteristics, with older patients and a more severe CHA_2_DS_2_-VASc mean score [44], showed an increased ICER to €3,625 per QALY gained for rivaroxaban, to €6,787 per QALY gained for dabigatran and to €40,864 per QALY gained for apixaban. The second scenario evaluated based on 10 years as the model time horizon, resulted in an increased ICER to €14,842 per QALY gained for rivaroxaban, to €13,670 per QALY gained for dabigatran and to €51,297 per QALY gained for apixaban. The analysis with a 20-year time horizon increased the ICER to €1,079 per QALY gained for rivaroxaban, to €4,696 per QALY gained for dabigatran and to €32,566 per QALY gained for apixaban. The third scenario conducted, considering HRs derived from Spanish RWE studies [27,45,46], yielded an ICER of €598 for rivaroxaban, of €21,986 per QALY for dabigatran and of €23,241 for apixaban.

**Table 4:** Scenario results.

	Incremental cost-effectiveness ratios/QALY (€)		
Scenario	Rivaroxaban	Dabigatran	Apixaban
	Increase vs VKA	Increase vs VKA	Increase vs VKA
**1: RWE population characteristics with older patients (77 years old) and a more severe CHA**_**2**_**DS**_**2**_**-VASc mean score** [44]	3,625	6,787	40,864
**2: Different time horizons** [44]			
10 years	14,842	13,670	51,297
20 years	1,079	4,696	32,566
**3: Treatment effect, in terms of event reduction, based on RWE studies** [27,45,46]	598	21,986	23,241

QALY, quality-adjusted life year; RWE, real-world evidence; VKA, vitamin K antagonist.

## Discussion

Although fewer MIs and strokes should be balanced with an increased number of bleeds, the results highlight that rivaroxaban and dabigatran are cost-effective versus VKA for stroke prevention in adult patients with NVAF in the Spanish healthcare setting. The reduction in clinical events such as stroke, the avoided costs associated with lower hospital admissions, lower rehabilitation proportion and no requirement of international normalised ratio monitoring, likely offsets the economic burden of NOACs. The results also suggest that rivaroxaban is the most cost-effective alternative; the level of stroke prevention results in a lower incremental cost and higher effectiveness versus VKA compared with dabigatran, as this is a key driver of the ICER. While apixaban was associated with fewer bleeds, the rates of MI and ischaemic stroke were simulated almost similar to VKA, as a result of quasi-neutral HRs. As per the high management cost of these two events, a strong impact on ICER is observed.

RCTs have already shown that NOACs are at least as effective as VKAs for stroke prevention in patients with NVAF [48–51], and existing cost-effectiveness analyses in literature have also found NOACs to be cost-effective in Spain [27,33,52] and in Europe [53–55]. Of note, Baron Esquivias et al. [33] showed that apixaban was cost-effective versus acenocoumarol in the Spanish healthcare setting, while the results of the present analysis are less favourable. However, the results of all these cost-effectiveness analyses, including the one from Baron Esquivias et al., were all based on treatment effects coming from clinical trials [27,33,52]. The disparities between these studies could be related to the appropriateness of the apixaban dose used in clinical practice, which may differ among the studies.

The main strength and added value of the present analysis is the full integration of RWE for the clinical input variables of the model. This provides more generalisable information on patient population characteristics and addresses several gaps related to the treatment effect. In real life, rivaroxaban was associated with a higher effectiveness than apixaban versus VKA for stroke prevention [26]. The difference in the results identified can be explained by confounding in a non-randomised comparison, but are more likely explained by an effect already well recognised in clinical practice: inadequate NOAC use at reduced doses is associated with a slightly better safety profile, but with a noticeable reduction in the effectiveness of stroke prevention [56,57]. Another explanation for these results could be that proposed by Fernández et al. 2020, who suggested that those NOACs with simpler dosage adjustment (by only one adjustment criterion–renal function, such as for rivaroxaban) could be correlated with less probability of dosage error by physicians and, therefore, with fewer thromboembolic events rates in real life [58]. This suggests that reduced doses of NOACs should only be used when indicated according to drug labelling and not when physicians perceive an increased risk of bleeding. Unfortunately, inappropriate drug use is frequent [59–61] and apixaban underdosing has worse effectiveness than other NOACs (rivaroxaban, dabigatran) in routine clinical practice [56,57].

The model structure of this cost-effectiveness analysis was based on the previous submission of rivaroxaban to the National Institute for Health and Care Excellence (NICE) in the UK [7], and although no major criticism has been raised by the evidence review group, several adjustments were implemented in order to integrate RWE. The initial population is based on an RWE study [21], to reflect characteristics of patients with AF in Spain. The progression of patients between states is done via transition probabilities derived from RWE, both for event rates for VKA, and for treatment effects [21–24]. The treatment effects were indeed taken from RWE meta-analyses performed for each drug separately versus VKA [26]. Costs and utilities were also derived from existing RWE studies [27,29–39]. It is worth noting that all key cost-effectiveness drivers of economic models submitted to NICE, including discontinuation rates, cost of international normalised ratio monitoring visits with VKA treatment, and patient baseline age were identified in RWE sources in the current model.

Several limitations must be presented. First, launched in Spain in 2016, edoxaban has not been assessed in the current analysis due to the paucity of RWE studies published in the literature so far. Second, the use of RWE in a meta-analysis may introduce a bias relative to residual confounding; however, this limitation was discussed previously and considered controlled, as per the stability of the results when scenarios were conducted [62]. Third, although all studies used to populate this model were drawn from RWE, Spanish sources could not be retrieved for all of them, but efforts were made to identify similar European studies. As an example, no MI rates for VKA and no utility values were available in Spain, and the inputs were drawn from a UK RWE study [23,29–31]. All three clinical experts agreed on this methodology where no Spanish data could be found and validated every input value taken from those European RWE studies. Lastly, gastrointestinal bleeding data were used as a proxy for major bleeding, but this assumption was acceptable in the rivaroxaban NICE evaluation [7].

## Conclusions

This RWE economic analysis suggests that rivaroxaban and dabigatran should be considered as cost-effective options versus VKA for stroke prevention in patients with NVAF in the Spanish healthcare setting. Rivaroxaban proved to be the most cost-effective alternative versus VKA, with an ICER of €952 per QALY gained. Rivaroxaban was followed by dabigatran with an ICER versus VKA of €4,612 per QALY gained, while apixaban resulted in an ICER versus VKA of €32,015 per QALY gained, which was above the cost-effectiveness threshold generally accepted in Spain. The results of this economic evaluation are reasonably robust, given the extensive sensitivity analyses conducted.

These findings provide valuable insights into real-world economic value of interventions, supporting the implementation of less restrictive use conditions for NOACs for stroke prevention in patients with NVAF in Spain.
